# Supplementary figures and images for: The genomic ecosystem of transposable elements in maize
Source: PLoS Genet. 2021 Oct 14;17(10):e1009768. doi: 10.1371/journal.pgen.1009768 (PMC8547701; doi:10.1371/journal.pgen.1009768)

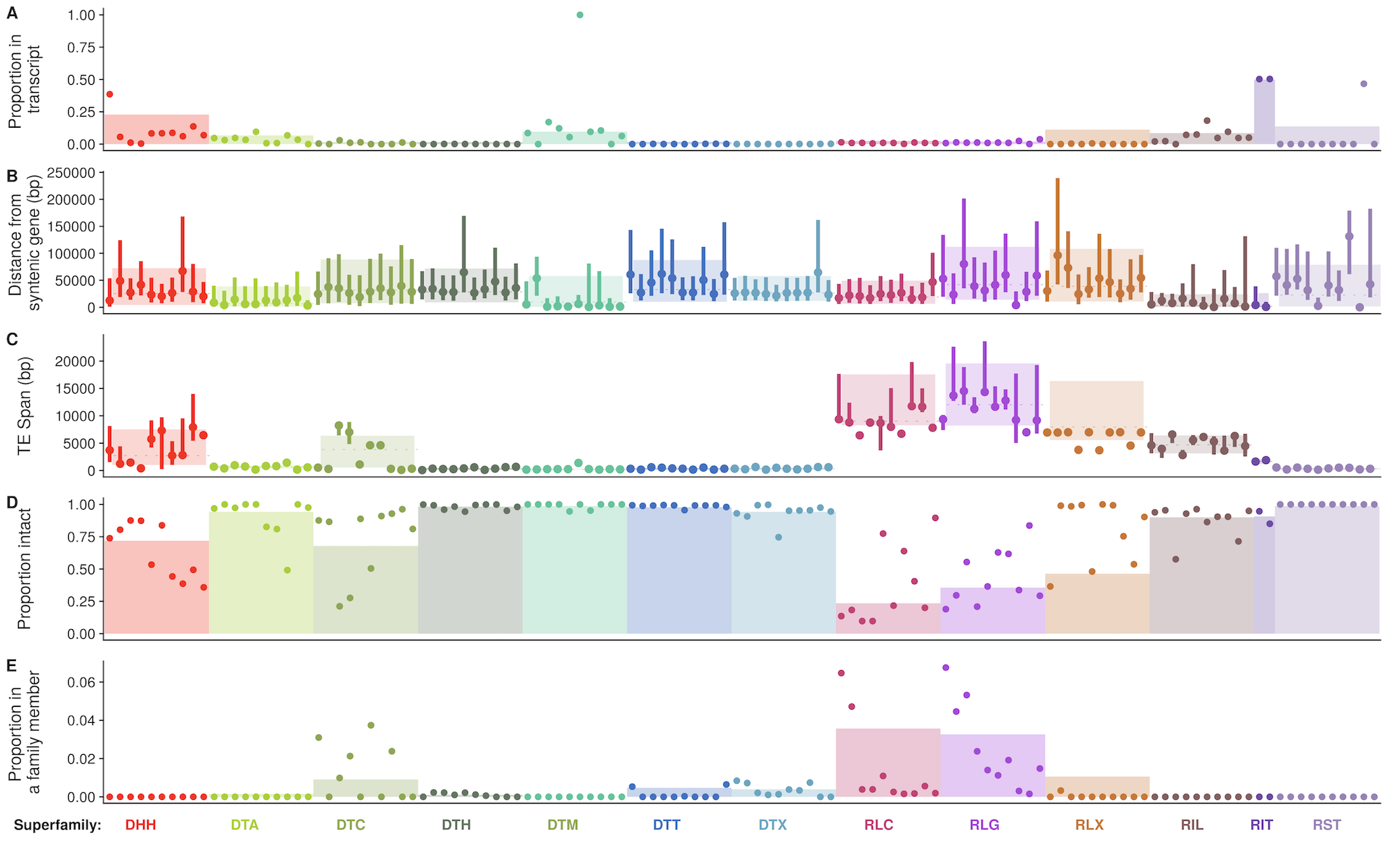

Supplement: S1 Fig — (A) Proportion of TEs within the transcript of a gene, including introns and UTRs. (B) TE span along the genome, summing both the base pairs of the TE and the base pairs of the TEs nested within it. (C) Proportion of TEs that are intact, that is, uninterrupted by the insertion of another TE. In (A and C), families are shown as points and superfamily proportions as a barplot, and in (B) families are shown with medians as points and lines representing ranges of upper to lower quartiles, with superfamilies shown as colored rectangles. (TIF) [file pgen.1009768.s001.tif]

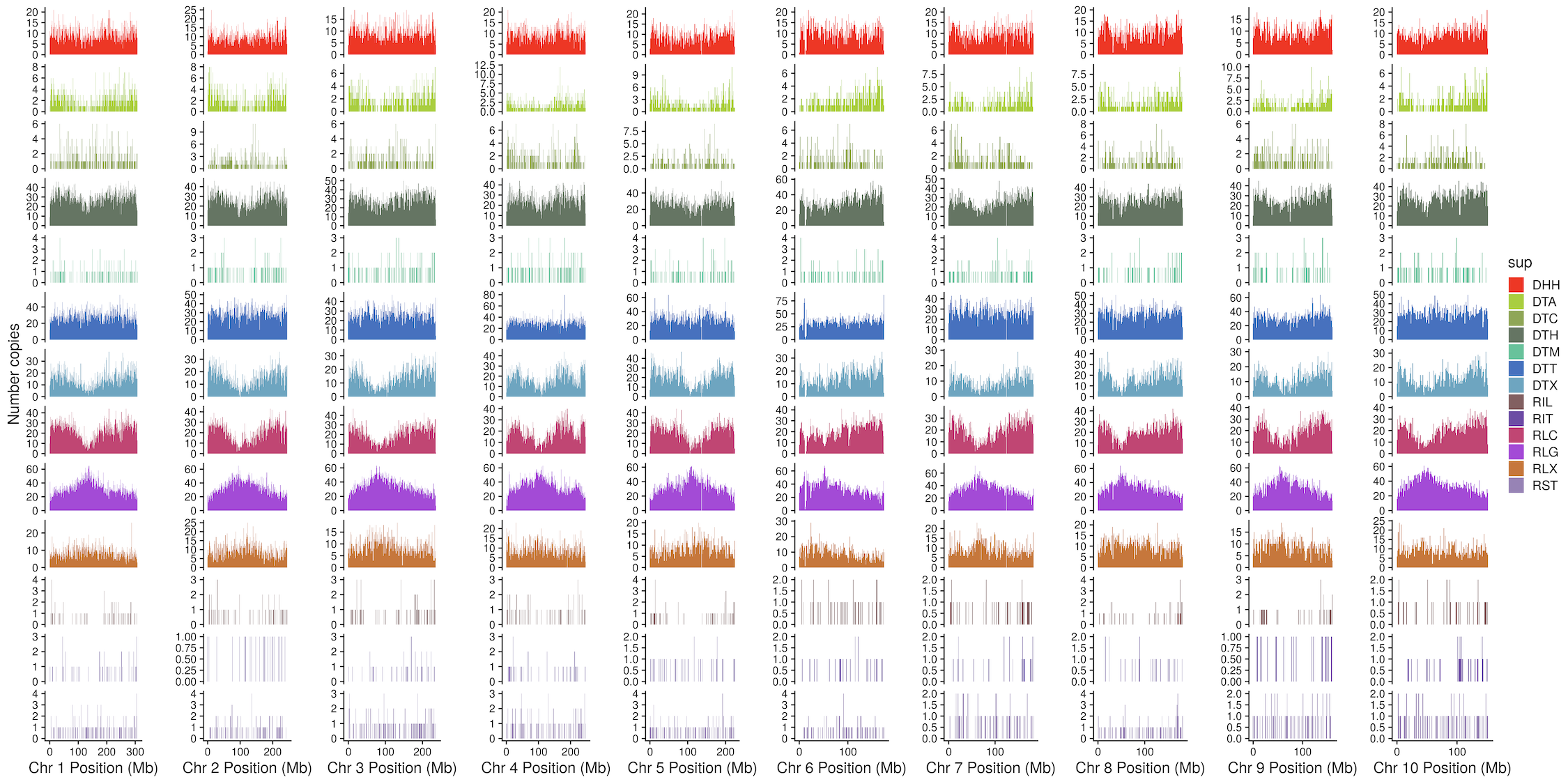

Supplement: S2 Fig — Count of TE copies of each superfamily in 1 megabase bins across each chromosome. (TIF) [file pgen.1009768.s002.tif]

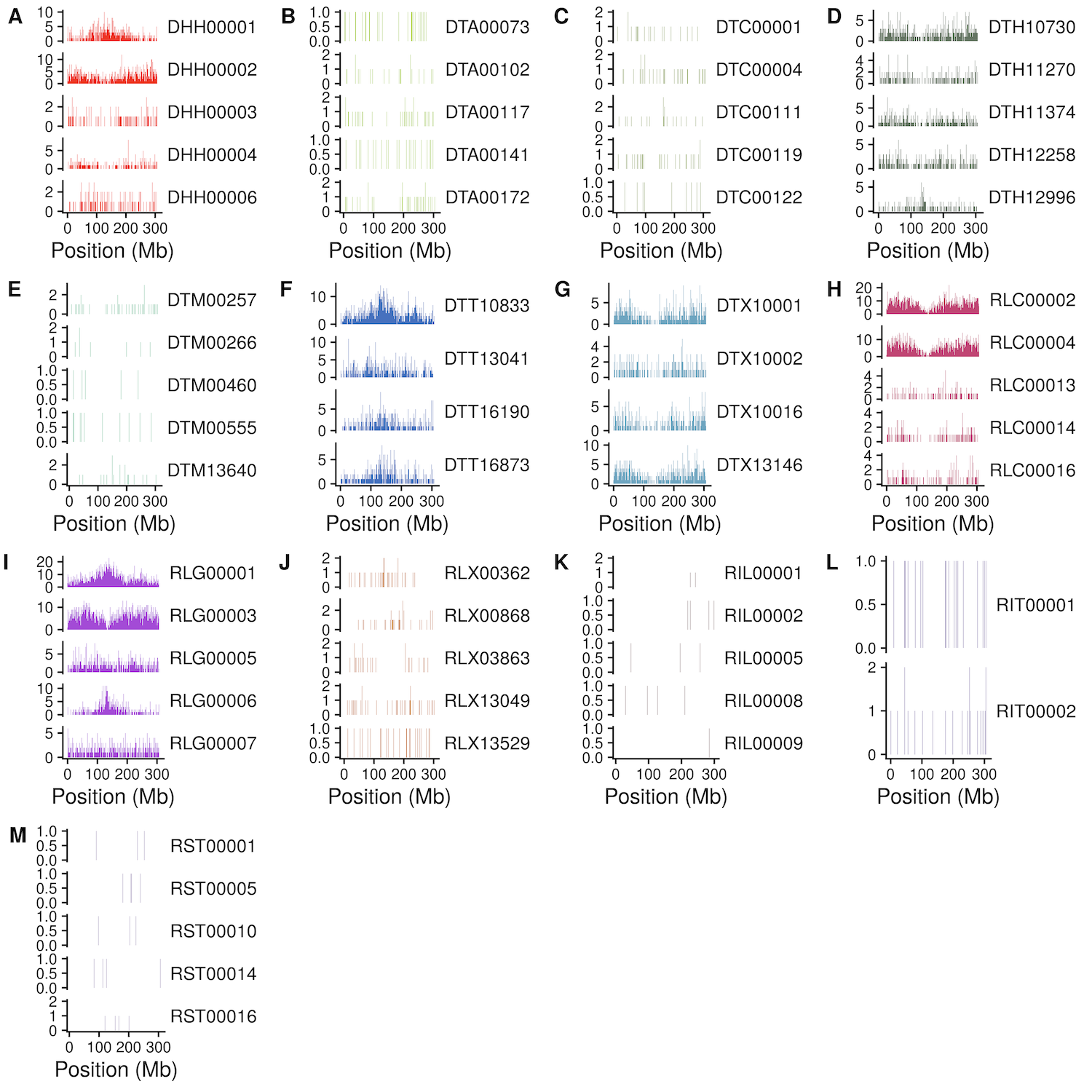

Supplement: S3 Fig — Count of TE copies in 1 megabase bins along chromosome 1. (A) DHH, (B) DTA, (C) DTC, (D) DTH, (E) DTM, (F) DTT, (G) DTX, (H) RLC, (I) RLG, (J) RLX, (K) RIL, (L) RIT, (M) RST. Note that some families have no copies on chromosome 1, including DTT10880 and DTX10177. Additionally, the RIT superfamily only has two families. (TIF) [file pgen.1009768.s003.tif]

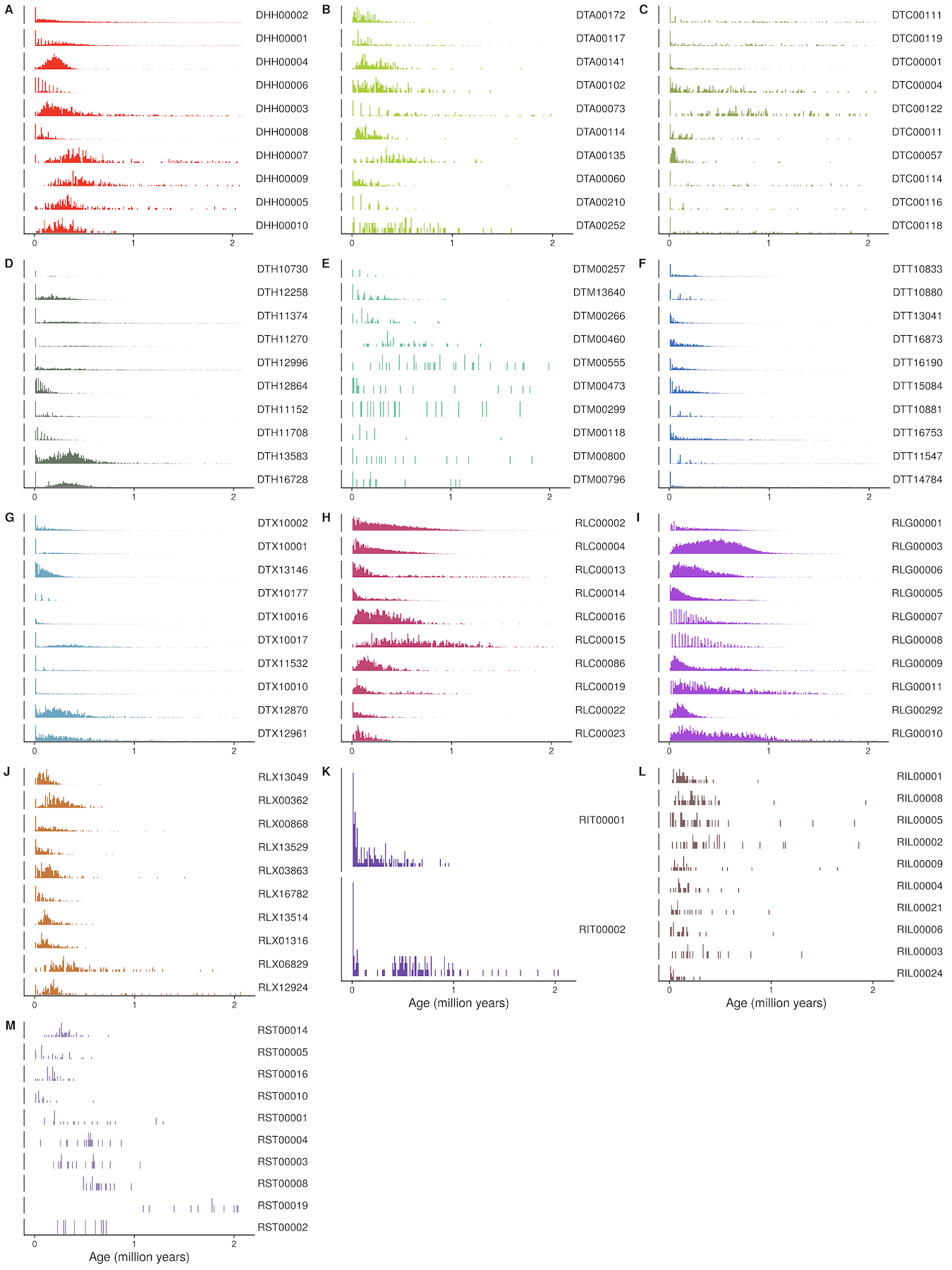

Supplement: S4 Fig — (A) DHH, (B) DTA, (C) DTC, (D) DTH, (E) DTM, (F) DTT, (G) DTX, (H) RLC, (I) RLG, (J) RLX, (K) RIT, (L) RIL, (M) RST. The RIT superfamily only contains two families. (TIF) [file pgen.1009768.s004.tif]

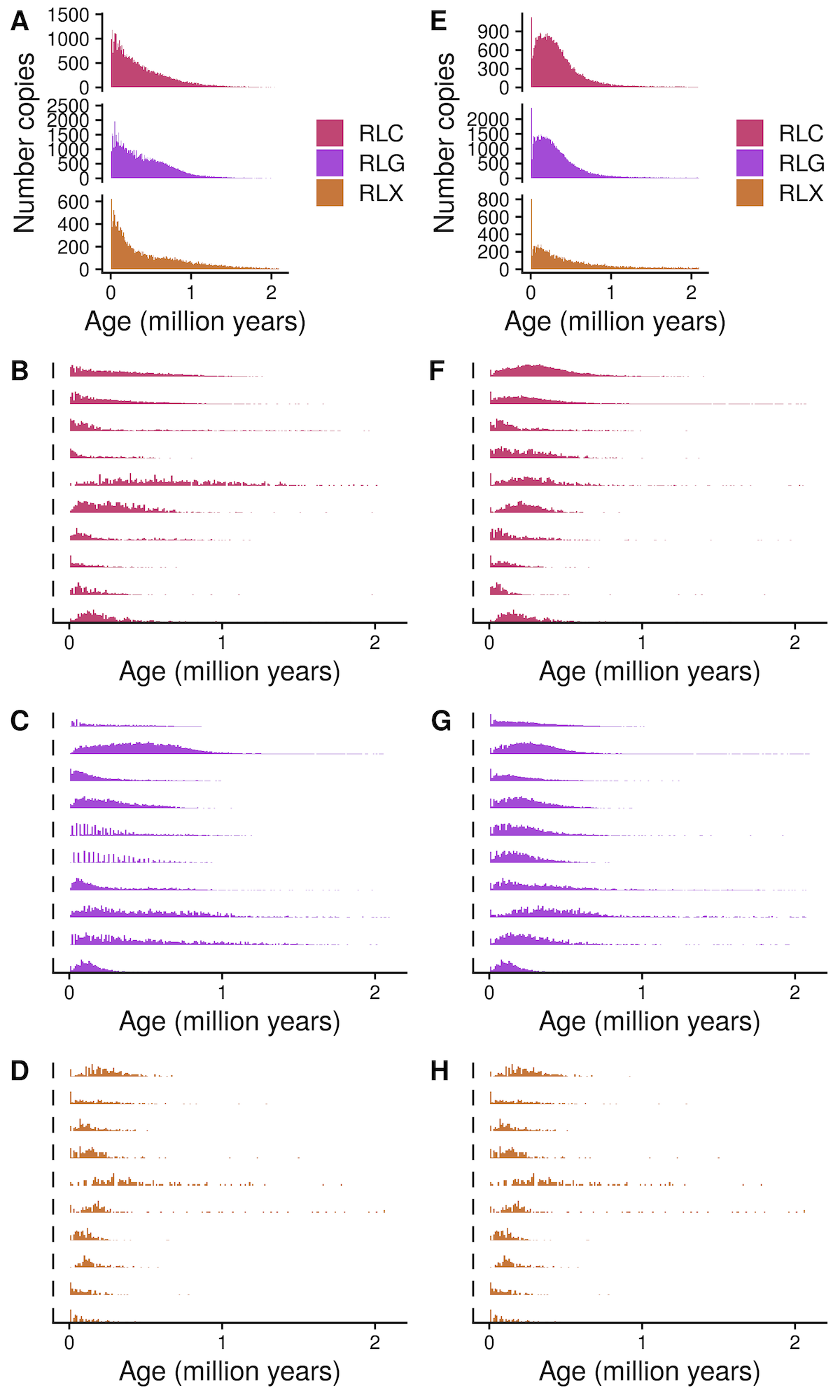

Supplement: S5 Fig — Ages in 10,000 year bins across each of the largest 10 families of each superfamily with at least 10 copies. Left plots (A-D) show LTR-LTR ages, right plots (E-H) show terminal branch length (TBL) ages. (A) all copies, LTR-LTR, (B) RLC families, LTR-LTR, (C) RLG families, LTR-LTR, (D) RLX families, LTR-LTR, (E) all copies, TBL, (F) RLC families, TBL, (G) RLG families, TBL, (H) RLX families, TBL. (TIF) [file pgen.1009768.s005.tif]

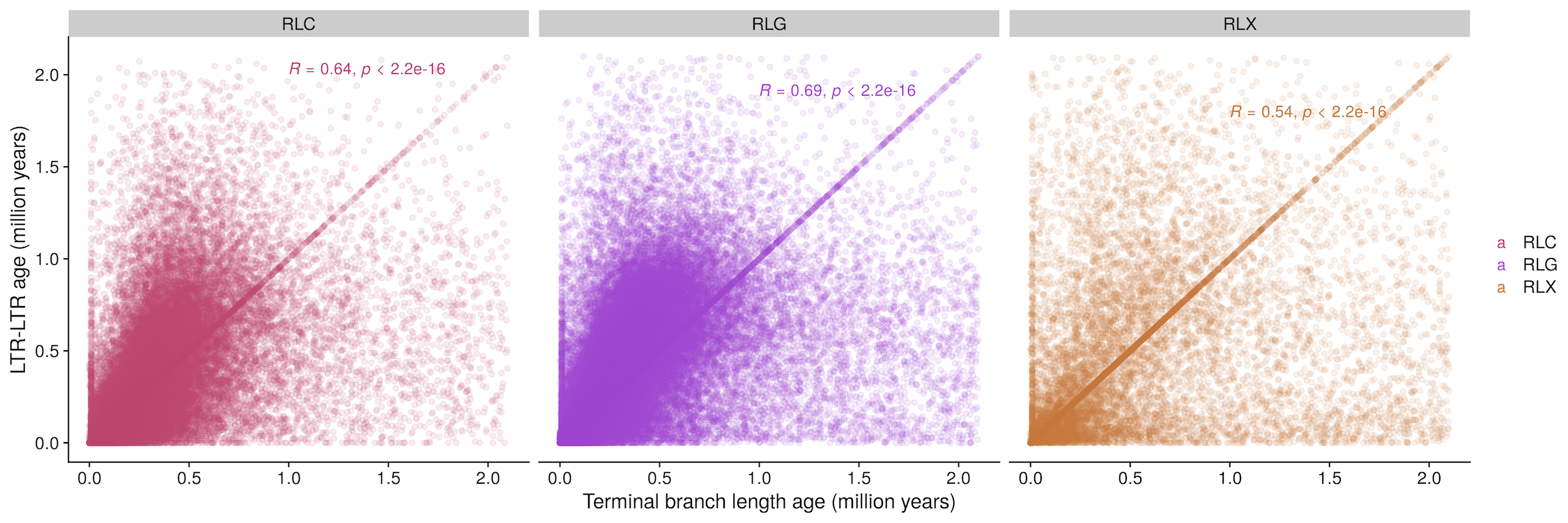

Supplement: S6 Fig — Spearman’s correlation coefficient shown on plot for each superfamily. (TIF) [file pgen.1009768.s006.tif]

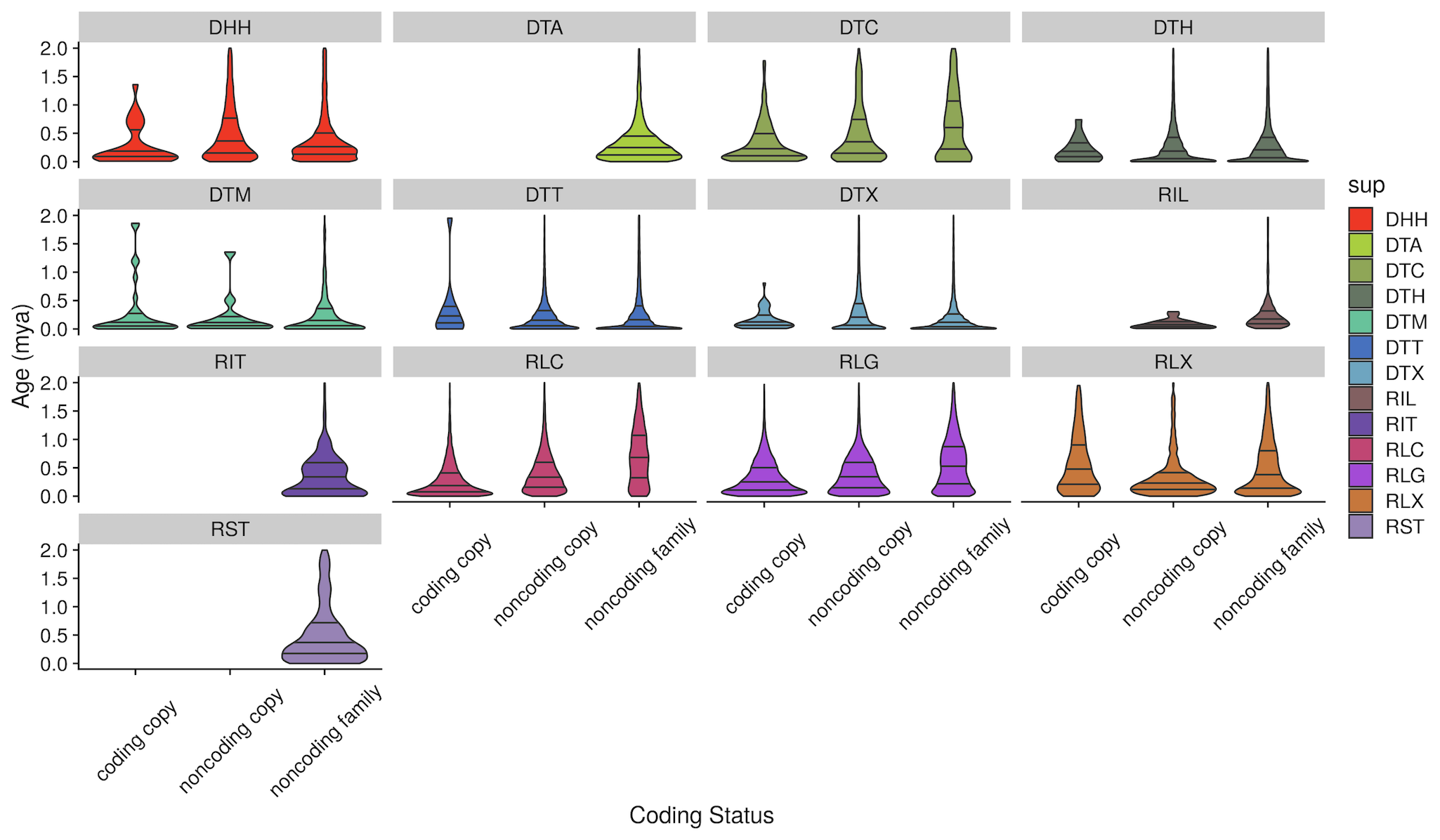

Supplement: S7 Fig — Violin plots with three lines, at median and 25th and 75th percentile. Only ages younger than 2 million years are shown. “Coding copy” refers to those copies that code for protein, “noncoding copy” refers to those copies that don’t code for protein, but a family member does, and “noncoding family” refers to copies from families without a coding member in B73. (TIF) [file pgen.1009768.s007.tif]

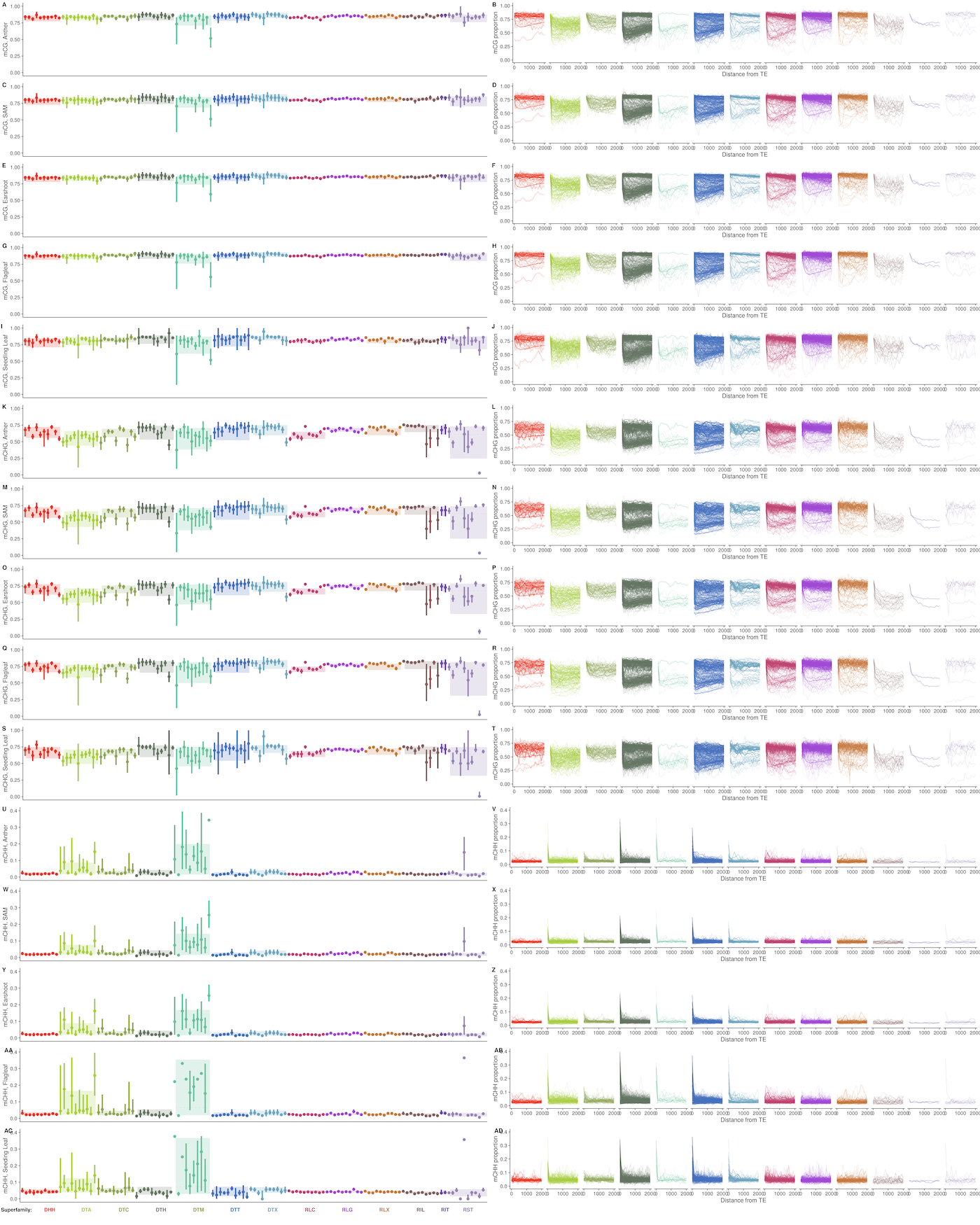

Supplement: S8 Fig — A-J: mCG; K-T: mCHG; U-end mCHH. Tissues on y-axis, from top to bottom: Anther, SAM (shoot apical meristem), Earshoot, Flagleaf, Seedling leaf. (TIF) [file pgen.1009768.s008.tif]

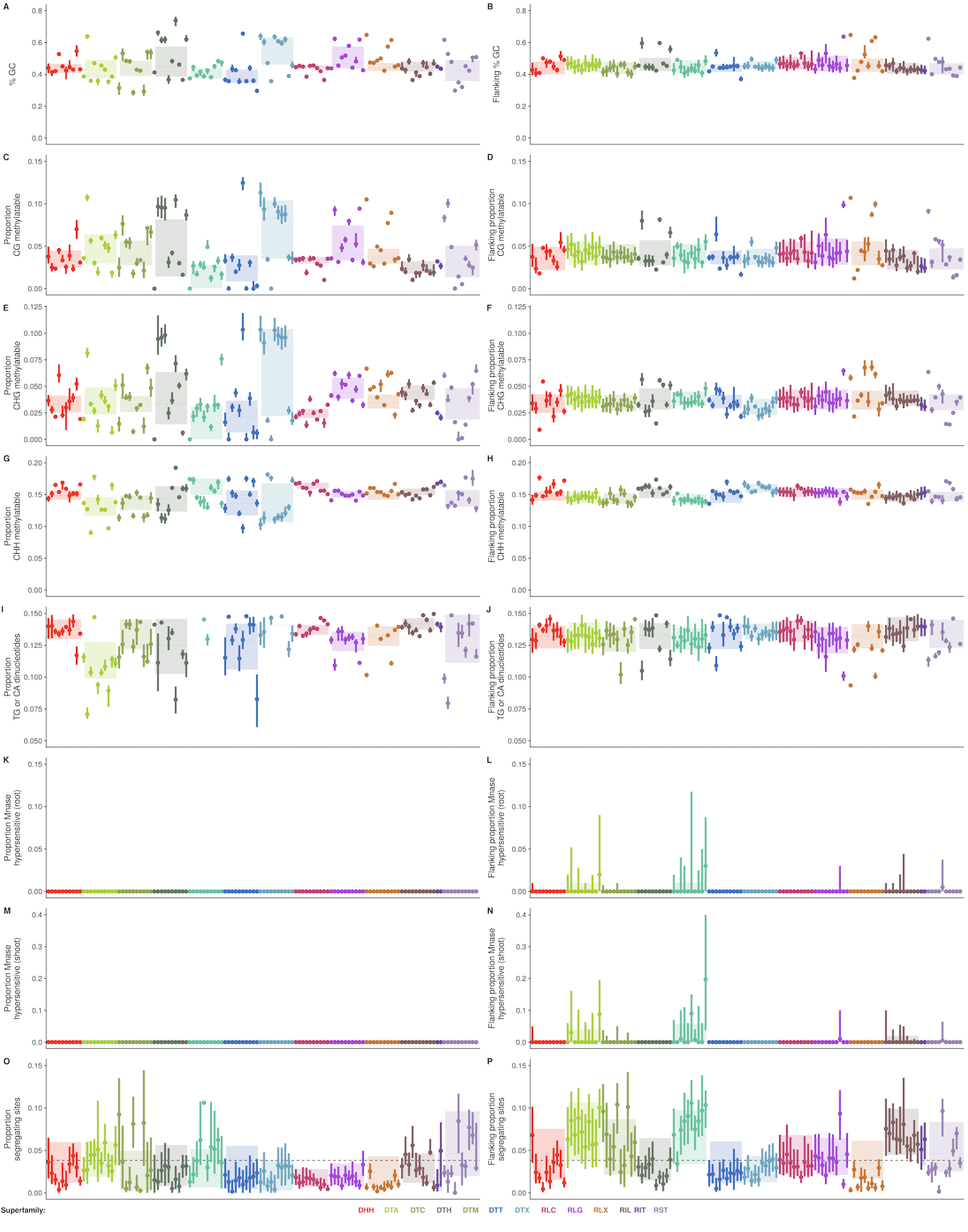

Supplement: S9 Fig — GC content in the TE (A) and 1kb flanking sequence (B). Proportion of sites methylatable in the CG context in the TE (C) and 1kb flanking sequence (D), methylatable in the CHG context in the TE (E) and 1kb flanking sequence (F), proportion of sites methylatable in the CHH context in the TE (G) and 1kb flanking sequence (H). Proportion of sites containing a TG or CA dinucleotide in the TE (I) and 1kb flanking sequence (J). Proportion of sites in MNase hypersensitive regions in root in TE (K) and 1kb flank (L), and shoot in TE (M) and 1kb flank (N). Proportion of segregating sites in the TE (O) and 1kb flank (P). (TIF) [file pgen.1009768.s009.tif]

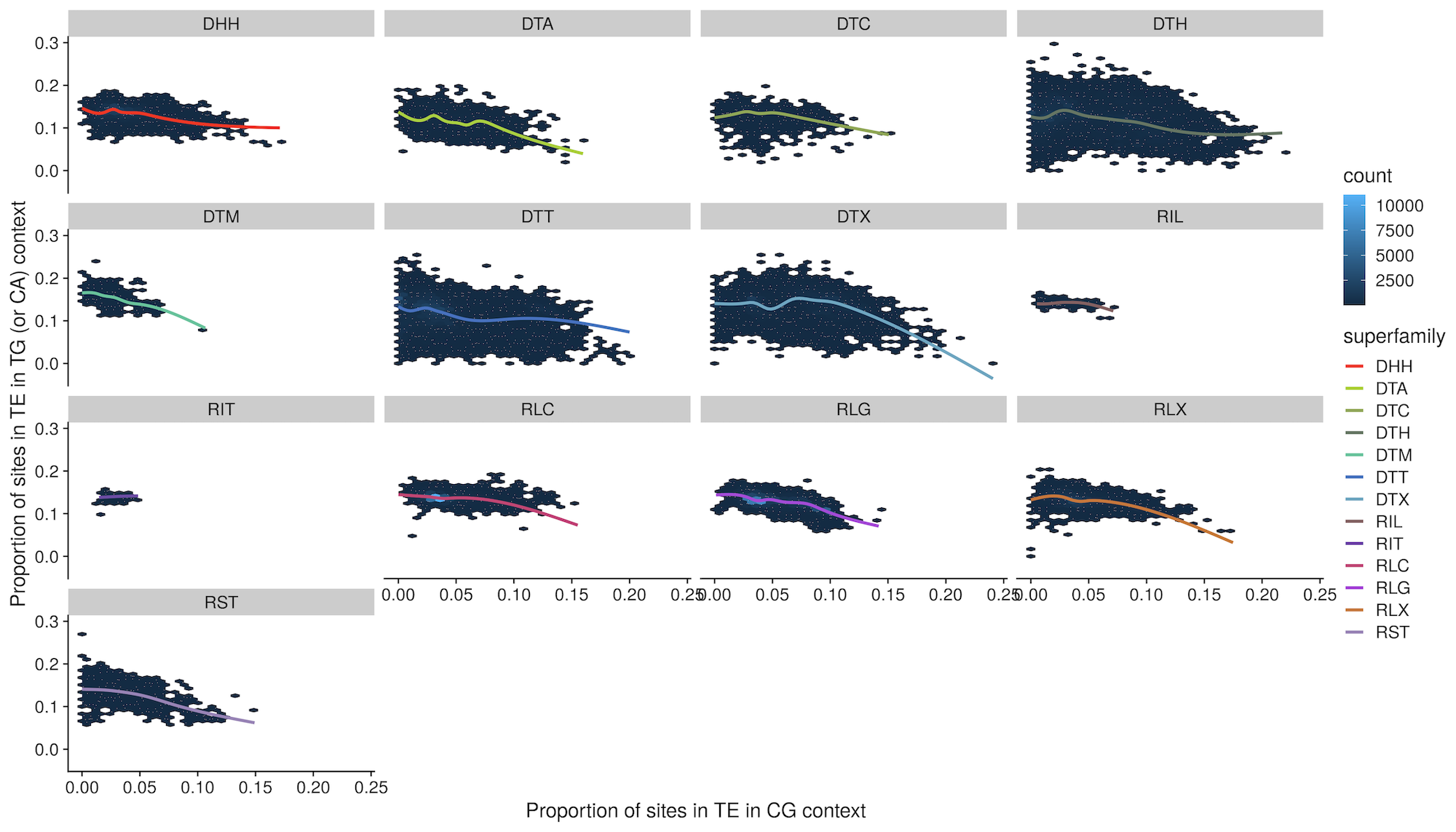

Supplement: S10 Fig — The x-axis reflects the proportion of cytosines in a CG context within the TE, and the y-axis reflects the proportion of dinucleotides in the TE that contain a TG or CA. (TIF) [file pgen.1009768.s010.tif]

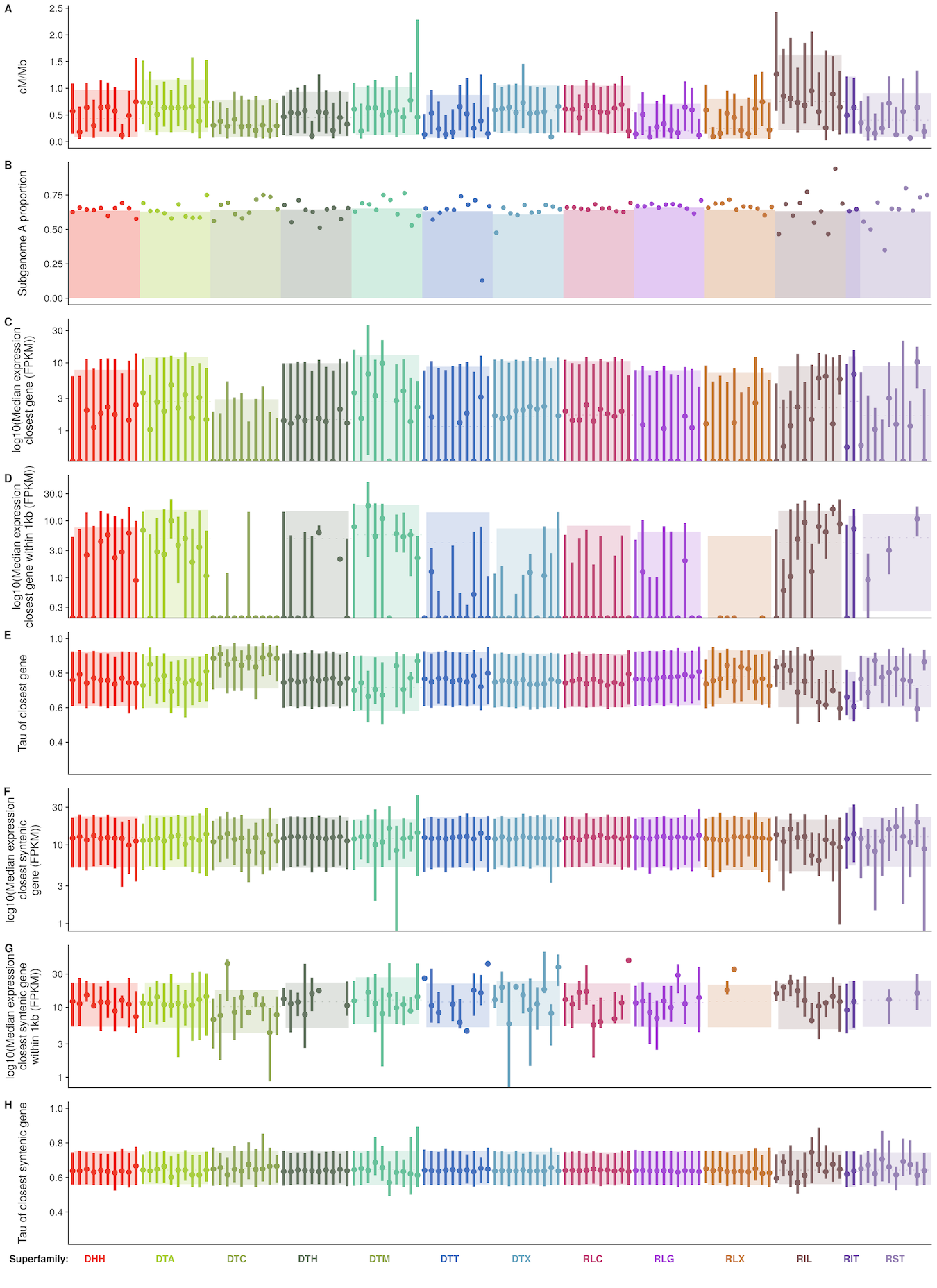

Supplement: S11 Fig — (A) Recombination rate across the TE, (B) proportion of TEs in subgenome A, (C) log10 median expression of the closest gene to each TE, (D) log10 median expression of genes within 1kb of the TE, (E) Tau of closest gene to each TE, (F) log10 median expression of the closest syntenic gene, (G) log10 median expression of closest syntenic genes within 1 kb, and (H) Tau of the closest syntenic gene. (TIF) [file pgen.1009768.s011.tif]

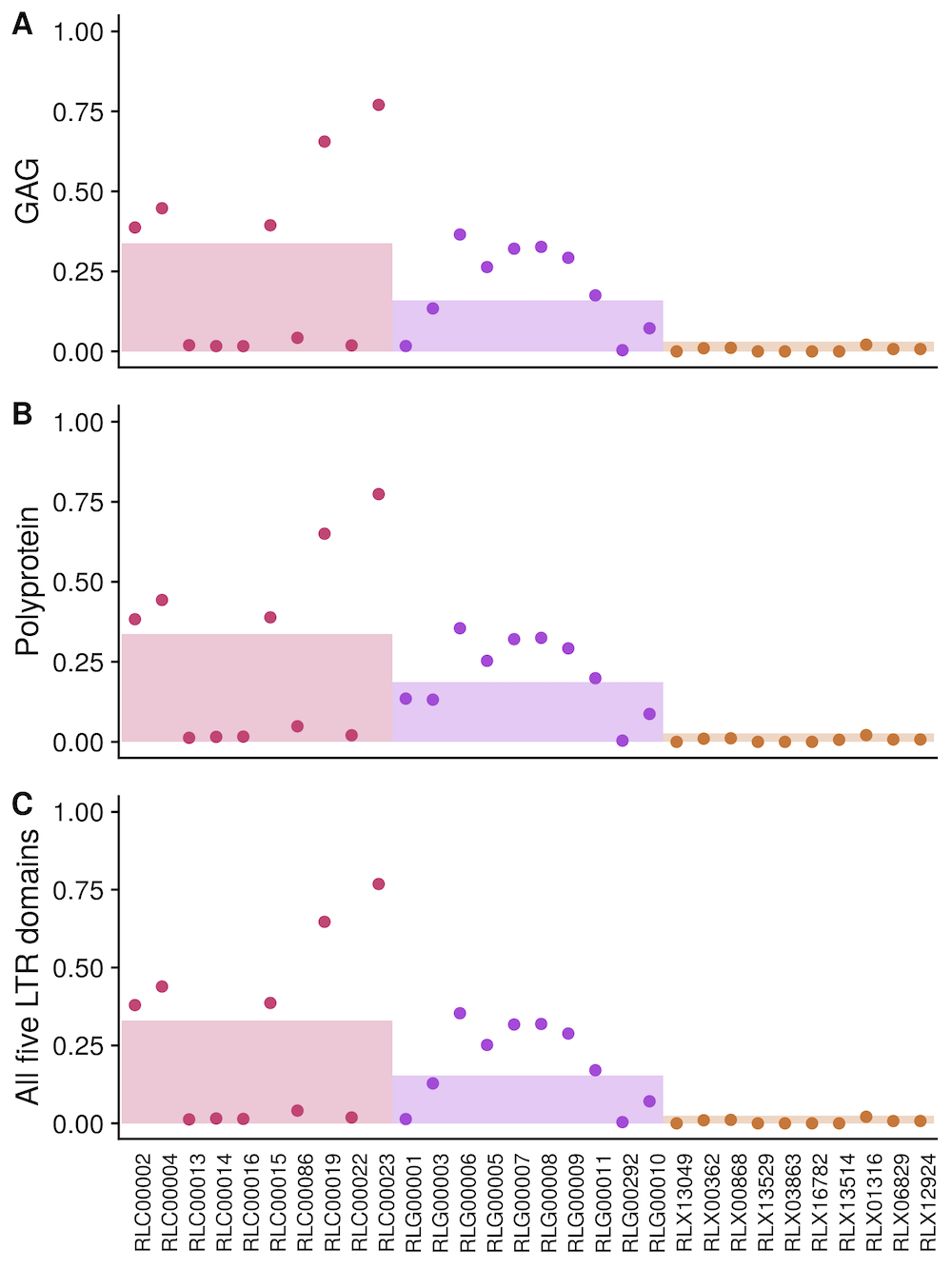

Supplement: S12 Fig — Shown are (A) the proportion of TEs with evidence of agglutination factor (GAG) domain present, (B) the proportion of TEs with evidence of all polyprotein domains present (aspartic proteinase, integrase, reverse transcriptase, and RNaseH), (C) the proportion of TEs with both GAG and Polyprotein present in the same element. Families are shown as points and superfamily proportions as barplot. (TIF) [file pgen.1009768.s012.tif]

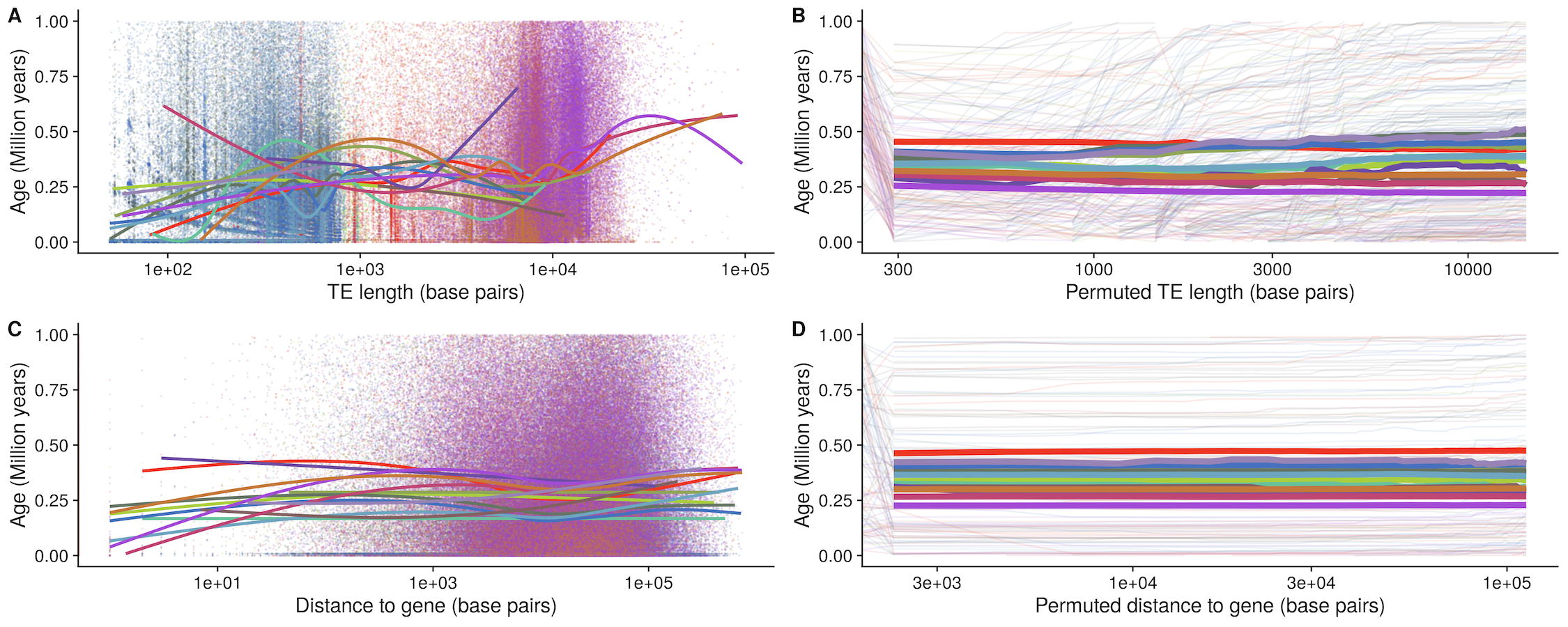

Supplement: S13 Fig — Raw relationship (A & C) and predicted relationship (B & D) of TE length (A & B) and distance to gene (C & D). (TIF) [file pgen.1009768.s013.tif]
