# Supplementary material for: The genomic ecosystem of transposable elements in maize
Source: PLoS Genet. 2021 Oct 14;17(10):e1009768. doi: 10.1371/journal.pgen.1009768 (PMC8547701; doi:10.1371/journal.pgen.1009768)
Supplement: S1 Text — (PDF) [file pgen.1009768.s020.pdf]

S1 Text

TE annotation methods

We modify the TE annotation from [1] to more fully capture DNA transposons. Briefly, 200 base pairs upstream and 200 base pairs downstream are extracted surrounding TARGeT matches [2] from searches using Maize TE Consortium (MTEC) exemplars [3] and detectMITE copies from Jiao *et. al* (2017) [1]. These are searched for perfect inverted repeats using RLibsTree [4], which define a candidate terminal inverted repeat (TIR). In order to allow for mismatches between TIRs, we search for a target site duplication (TSD) outside of this region. TSDs are searched sequentially in three ways:

1. Use the length of the TSD expected for the superfamily to look at sequence immediately adjacent to the longest identical TIR match, and see whether these adjacent TSD sequences are identical.
2. Resect the TIR by 1, 2, and 3 bp, to see if there is an adjacent TSD. For example, a TSD that is TTA will extend the TIR by a single base pair, even though that additional T/A actually belongs to the TSD.
3. If there is not a match to the previous rules, search for a TSD in the 20 base pairs adjacent to the longest identical TIR, then extend the TIR internal to this.

For these new TIRs, count the sequence distance between the two 'new' TIRs. If this distance is $\leq$0.2, keep the TE and consider these the TIR and TSD. This generates mismatches between TIRs.

Supporting code available at `https://github.com/mcstitzer/annotate_TIR`.

Overlapping TEs are then filtered, as in [1, 5], allowing nested copies but trusting overlapping copies with incompatible boundaries of LTR >TIR >Helitron.

References

1. Jiao Y, Peluso P, Shi J, Liang T, Stitzer MC, Wang B, et al. Improved maize reference genome with single-molecule technologies. Nature. 2017;546(7659):524–527.
2. Han Y, Qin S, Wessler SR. Comparison of class 2 transposable elements at superfamily resolution reveals conserved and distinct features in cereal grass genomes. BMC genomics. 2013;14(1):71.
3. Schnable PS, Ware D, Fulton RS, Stein JC, Wei F, Pasternak S, et al. The B73 maize genome: complexity, diversity, and dynamics. Science. 2009;326(5956):1112–1115.
4. Lang DT. RLibstree: Suffix Trees in R via the libstree C library) - `https://github.com/omegahat/Rlibstree/`; 2019.
5. Springer NM, Anderson SN, Andorf CM, Ahern KR, Bai F, Barad O, et al. The maize W22 genome provides a foundation for functional genomics and transposon biology. Nature genetics. 2018;50(9):1282.
