# Supplementary material for: The genomic ecosystem of transposable elements in maize
Source: PLoS Genet. 2021 Oct 14;17(10):e1009768. doi: 10.1371/journal.pgen.1009768 (PMC8547701; doi:10.1371/journal.pgen.1009768)
Supplement: Attachment — Submitted filename: ecology_of_the_genome_RevisionResponse.pdf [file pgen.1009768.s021.pdf]

From the editor:

Should you decide to revise the manuscript for further consideration here, your revisions should address the specific points made by each reviewer. In particular, the revision should address the specific comments of Reviewer 1 regarding clarity of figures, description of transposable element taxonomic categories for the general reader, and statistical support for some assertions. Additionally, both reviewers point out that the "genome ecology" concept, while intriguing, is largely employed in this manuscript as an analogy rather than a mode of inference. This should be addressed in a revised manuscript. We will also require a detailed list of your responses to the review comments and a description of the changes you have made in the manuscript.

Response to the reviewers:

We thank the reviewers for these helpful comments. We have revised the manuscript to better highlight that we're making ecological analogies, and not explicitly using ecological models. We went this direction to prevent adding more material to an already dense paper. We've tried to simplify some of the TE jargon when introducing classes, orders, superfamilies and families of TEs, and highlight their identities within figures. We've added more descriptive and intuitive legends to all figures, and moved non-essential sub-figures to the supplemental figures. In the following pages, we address reviewer concerns point by point.

Reviewer 1

Reviewer Point P 1.1 — This is a "bird's eye" view of transposons in the maize genome. It makes the assertion that the maize genome constitutes an ecosystem, in which different families of elements occupy distinct niches. Certainly, the shear diversity of elements with respect to their location, methylation and expression supports this idea. I'm not sure the authors really support the idea that elements actually interact with each other, which weakens the argument. For instance, evidence that one family actually negatively effects the activity of another family would support the hypothesis, as would evidence that some elements tend to insert into other elements rather than themselves. However, evidence of cooperation between families (Cinful and Zeon) is intriguing. Based on the data supplied here, I would suggest that the genome ecology hypothesis is, at this point, mostly a useful metaphor, and a good place to start for further analysis. As indicated below, I think presentation of the data in the figures could be improved for greater clarity. If I was confused, I'm guessing other readers will be confused as well). I would have also liked to have the definitions of superfamilies and families more precisely defined, and I would have liked to have had instance in which the variation observed really does support and ecological model. Overall, because the subject is so broad (hard to believe when we are looking at a single genome!), I think the authors did a pretty good job, particularly since all of the raw data and code has been made available. I very much hope that this information is integrated into TE models in the current maize genome version, since it's a mess right now. Each section could (and likely will be) a manuscript on its own, but this manuscript opens doors for numerous other, more in depth analyses.

Reply: We agree that as presented, this is predominately metaphorical ecology, and rewrote sections in the introduction and conclusion to highlight this. We opted not to introduce explicit models, as we struggle with the trade-offs in clarity between exhaustively examining hypotheses arising from pairwise relationships between families and over-generalizing patterns. We hope in future studies to more explicitly test ecological models, especially to track the robustness of ecological patterns across individuals with the growing availability of multiple maize reference genomes from diverse germplasm.

Reviewer Point P 1.2 — Line 28. That's a pretty big claim. The manuscript should support it, but I'm not sure it does.

Reply: This comment refers to the statement 'TEs that coexist in time and space compete and cooperate with each other.' We agree that we present limited direct evidence of species interactions between TE families, so removed this clause from the abstract.

Reviewer Point P 1.3 — Line 41. A quibble, but maybe say maize genome?

Reply: Replaced '85% of DNA in a maize plant' with '85% of DNA in a maize genome.'

Reviewer Point P 1.4 — Line 93. As far as I know, this is still a hypothetical mechanism for eukaryotic Helitrons, since no one has demonstrated this mechanism in these organsism. Best to say, "are thought to..."

Reply: Good point, added 'are thought to.'

Reviewer Point P 1.5 — Line 176. It might be useful to remind the reader as to how orders and superfamilies are defined. Just a couple of sentences for those not familiar with the terminology.

Reply: We added full names for each superfamily at this position in the start of the results. We also added text to the introduction that highlights that protein domains encoded by the TE or a family member are the defining features of superfamilies.

Reviewer Point P 1.6 — Line 190. Typo.

Reply: This was intended to be a link, but it didn't render properly. Fixed.

Reviewer Point P 1.7 — Line 209. Not sure it's worth doing, but it would be interesting to know if some TEs are more attractive targets for insertion - this would fit into a competition model. For instance, do some TEs avoid insertions into themselves? That would be kind of cool.

Reply: We've included 'insertion into family member' and 'insertion into a superfamily member' as new features here to begin to address this. There are only five families (greater than 10 copies) where more than 10% of individual TEs are inserted into a family member (Supp. Figure 1E). Early studies of maize LTR retrotransposons suggested that for those copies that are nested into another copy, they are 5x more likely to insert into LTRs rather than internal regions (SanMiguel *et al.*, 1996; Bennetzen, 2000). Some surveys of Drosophila TEs (e.g. Inouye *et al.* (1984)) predict exactly the opposite - new copies target their own family, essentially inactivating the older member and assuring reproduction of the newly inserted copy. We're not sure what's happening here, but like this addition as something that invites future study.

Reviewer Point P 1.8 — Line 213. Again, it sure would be interesting to see if there are any biases in insertions. I'm not trying to make more work for the authors, but this would be really interesting. Also, give my admittedly anecdotal evidence, I'm surprised that these TIR elements are not closer to genes.

Reply: We agree, and have added the family- and superfamily- insertion metric noted above.

We also were surprised by the elevated distance of TIRs from genes. Much of this comes from the large number of new TIR families described in this annotation. Early annotations of maize TIR elements relied on sequence from BAC contigs. The BACs selected for sequencing were enriched for gene content (Schnable *et al.*, 2009), which may have enriched for TE families which insert near genes. When we subset to the 893 TIR families from the initial genome assembly that are found in the Maize TE Database (Schnable *et al.*, 2009), we recapitulate a closer relationship of TIR elements to genes (1.6 kb median distance for all genes, 4.0 kb for syntenic genes). We've added a sentence to the discussion that contains our explanation for the difference as presented here.

Reviewer Point P 1.9 — Figure 2. For the non-specialist, the names in the figure don't mean much, so it is hard for them to get the major point, other than length of different TEs varies. Perhaps it would be worth adding something in the legend saying, "Element designations beginning in a DT represent TIR DNA elements, those beginning in a DH are helitrons," and so on. The same perhaps in the other Figures with these designations? I know it's redundant, but I think it's always a good idea to keep the reader oriented, so that if they see an exceptional family, they will know right away what kind of element it is.

Reply: Thank you for this helpful suggestion! We added text to the legend highlighting how orders and classes can be deduced from the superfamily name. In other figures, we replaced legends with superfamily names on the x axis, and added bars representing orders and classes below those names for figure 2 where the figure style is first introduced.

Reviewer Point P 1.10 — Figure 3. It is very difficult to match the color designations below with the graphics above. In addition to the label, I would go ahead and individually label each tract (particularly for color blind readers, this would be a big help).

Reply: Agreed, replaced all legends that designated superfamily with superfamily names on the x or y axis.

Reviewer Point P 1.11 — Line 219. Again, a reasonable claim is made concerning length, but you could do the math and see if that is the case. That is, does is the length of elements the sole determinant of whether or not it has an insertion into it?

Reply: Across all TEs, this is true, see figure.

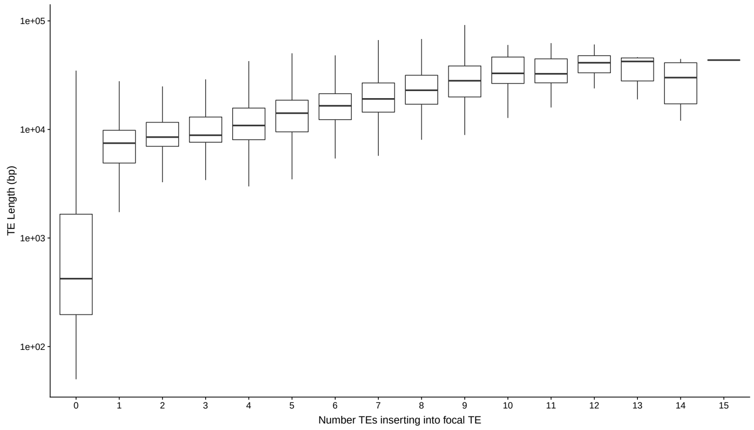

Fig. 1. Longer TEs are disrupted by more insertions TE length on the y-axis is presented on a log10 scale, the number of TEs nested into the focal TE is shown on the x axis.

We couldn't find a place to fit this comparison into the main text, but if reviewers think it is necessary, we can do so. We're introducing length and proportion disrupted for each order at this point in the text, and felt that although interesting, this comparison added too much detail.

Reviewer Point P 1.12 — Line 233. "Arrived in the genome?" Does that mean that they were horizontally transferred from another species? Do you mean that a given copy of these elements at a given position is 350,000 years ago?

Reply: We see how this language was unclear. This means inserted at the position we observe them today (from families already present in the genome). Changed the text to 'have a median age of 350,000 years.'

Reviewer Point P 1.13 — Line 249. Previous analysis has suggested that the average distance of a TSS to a TE is roughly 300 bp (with a lot a variation). On net, these distances seem much farther. Do you have a calculation of the average distance upstream of TSSs of TEs?

Reply: We agree that these distances might seem far, but we are presenting distances from the perspective of each TE. Most of the time, the closest gene to each TE requires passing through other TEs in the genome to reach that closest gene. To make the comparisons noted by the reviewer, we calculated these distances from the perspective of a gene. The median distance of each gene to the closest TE is 496 bp. This is further than previously suggested, but we believe this may be affected by limitations of the v4 maize gene annotation. UTR

features are poorly characterized in the gene annotation of v4 of the maize genome (Washburn *et al.*, 2019), leading to a systematic positioning of TSS'es at the start of the CDS, especially for genes that lack an intron. Unlike the cited study, we are hesitant to go back to a previous version of the genome to remedy this, because we'd lose the improved contiguity of repeat regions in the PacBio-based v4 genome. We believe that further progress in gene annotation and ongoing experiments that capture the 5' ends of transcripts can more accurately address this question. Additionally, 5' UTRs are on average 256 bp in the maize genome, suggesting an intuition of ≈ 300 bp is not that different from our findings (admittedly, this UTR length comes from the problematic v4 gene annotation, but excludes genes with absent UTRs).

Reviewer Point P 1.14 — Line 254. It might be useful to remind the reader as to how families are defined.

Reply: Added sentence describing family identification at the start of the 'Features of TE Families' section.

Reviewer Point P 1.15 — Line 289. "estimated with LTR-LTR" should be "estimated using LTR-LTR". Also, if you are going to say this, then how are the age of the other elements established? I'm assuming by divergence from the consensus sequence of a given family. Perhaps it would be a good idea to reference your materials and methods here, since dating TEs is a non-trivial task.

Reply: Changed wording here, added reference to Materials and Methods. We added a sentence to the beginning of the paragraph explaining how we're using terminal branch lengths of TE phylogenies to estimate age for all TEs.

Reviewer Point P 1.16 — Line 294. Well, yes and no, since you are looking at a combination of any restrictions and allowances and selection following insertion. It's important that this distinction is made clear.

Reply: Agree that this is misleading, and added a clause about survival after insertion as suggested.

Reviewer Point P 1.17 — Line 302. Which proteins? Those that are known to be functional (hAT, CACTA and MULEs have functional transposases) and proteins that are full length and are potentially functional?

Reply: We're using hmm profiles for transposases, integrases, and replicases from JCVI GenProp1044 and PFAM PF02689, PF14214, PF05970 to find homology to TE proteins. We're asking whether the copy contains *any* coding capacity that matches a known transposase, reverse transcriptase, or helicase, which we admit is weak evidence for anything approaching autonomy. We're hoping that the conservation of DNA binding at the transposase catalytic domain allows us to extend protein matches to other TIR superfamilies. We considered using catalytic domains from Yuan and Wessler (2011), but couldn't make decisions about whether to exclude non-plant eukaryotic superfamilies. We struggle with the fact that after millions of years of surviving in the maize genome, there is very little expected conservation of TE sequence, including protein coding sequence. That is, after insertion, there's not necessarily natural selection to maintain function of the protein coding sequence (although there likely is for younger, actively transposing copies). We've added more caveats into the results and discussion of protein coding sequence to try to temper our presentation of these results.

Reviewer Point P 1.18 — Line 315. Confusing wording. 0.6% of families within a super family (implied by the wording), or 0.6% of elements within each family?

Reply: 0.6% of all TIR families (across all TIR superfamilies) have at least one copy that matches a transposase. We've split the sentence in two to separate LTR retrotransposons from the rest, which hopefully helps clarity since the number of protein domains needed for LTR retrotransposition is higher than the other orders listed.

Reviewer Point P 1.19 — Line 319. Should read "potentially autonomous elements". Autonomy is a functional designation.

Reply: Completely agree, changed.

Reviewer Point P 1.20 — Figure 5. As with the other figures, this figure could be more clear. In 5A and B, since each dot has a range around it, I'm assuming there are multiple proteins represented. Are these families (i.e. RLG0001)? And for each color, all of the families within each superfamily that encode a protein are shown? I get it now, but I think the more explicit you can be the better. In 5C, "Presence of GAG, all five domains (GAG and Pol), and Pol (which encodes four domains) in LTR retrotransposons." So, GAG has five domains that includes a Pol, and Pol (a different Pol?). A bit confusing. Why are all the superfamilies listed with and without colors? What is the point of the list in which they are black? In 5G, it's not clear how the two axes are organized. Is the X axis organized by similarity of patterns of expression? If so, how is the Y axis organized? The color bar on the left seems to suggest that patterns of expression are more or less randomly distributed, but other than that, I'm not sure how informative this is. And what is "sup"?

Reply: We've restructured this figure extensively. We moved the former 5C to the supplement, to use the figure legend to better describe what's going on. We removed the extraneous legend. We added text to the paper to do a better job of guiding through the main finding of the former 5G — that most TE families are not expressed in any tissue, and there are groups of families expressed in a tissue-specific manner.

Reviewer Point P 1.21 — Line 354. Maybe or maybe not, since you are dividing the expression level by the total copy number, so higher copy number elements will inevitably show lower per element expression levels. It's certainly possible that a small subset of a large family is expressing at a relatively high level. It might have been useful to only look at RNAseq data that maps perfectly to intact elements (good tsds, good ORFs), but there are problems with this as well.

Reply: Yes, we have struggled with quantification of TE transcripts. We rely on our approach from Anderson *et al.* (2018, 2019) that summarizes multiply mapped reads at the level of the family. This prevents to some degree systematic biases that might occur when only looking at uniquely mapped reads. For example, young families with recent expansions (and therefore high sequence similarity) might actually be expressed at high levels, but relying on uniquely mapped reads would mean we consider them not expressed. Almost certainly for large families, it's highly unlikely that all copies are being transcribed at low levels, and instead reflects a few highly expressed copies. We've added description to the text with these caveats. We're leaving this analysis as-is in light of these issues, but would be welcome to additional suggestions.

Reviewer Point P 1.22 — Line 372. It is well known that tissue specificity in pollen is likely due to transient relaxation of silencing in the vegetative nucleus and is related to potential activity rather than actual activity. The tough part of this analysis is TEs are often transiently expressed in response to stresses, and which is not represented in this panel. Actually, if data existed for stressed plants in a subset of tissues, you might have gotten some interesting data. As it is, I think the most you can say is that most TEs, under normal conditions, are probably expressed at very low levels.

Reply: Agree with these points, but analysis of stress data was outside the scope of the paper. Stress data sets exist, and we have analysed them elsewhere (see Anderson *et al.* (2019)). We hope our cursory analysis encourages future work that focuses on disentangling the signal of single element expression from family level expression, especially under different stress conditions.

Reviewer Point P 1.23 — Line 397. Did the level of methylation correlate with expression level? Also, it is worth pointing out the CHH methylation is largely restricted to TEs adjacent to genes, so position, rather that the type of element can be important. If a given family (e.g. MULEs) are closer to genes, they are more likely to have CHH methylation. This may or may not have to do with how effectively they are silenced.

Reply: In light of Reviewer 2's requests for differentiating results and discussion, we've added CHH methylation interpretations later in the discussion. A deeper dive into methylation and its effects on nearby genes is provided

in Noshay *et al.* (2019), which focuses on a subset of abundant TE families to look specifically at methylation and how the spread of methylation from certain TE families can impact genes. We've added citations to Noshay *et al.* (2019) to direct readers to that more methylation-focused paper.

Reviewer Point P 1.24 — Line 399, 400. typos.

Reply: Fixed - citations didn't render.

Reviewer Point P 1.25 — Line 399. I don't know what this means. Preferential insertion based on methylation?

Reply: Yes, citation that we meant to have there is (Sultana *et al.*, 2017), which highlights ways in which TEs target their own insertion based on DNA and histone methylation. We've reworded this sentence to reflect what we really mean — TEs targeting their insertion to heterochromatin.

Reviewer Point P 1.26 — Line 400. Or that they insert into regions that are already methylated. How many of the flanking sequences are methylated because they are TEs? For many of these flanking sequences, the only way to know for sure if the TE is causing an effect is if it is polymorphic. I believe the W22 methylome and sequence is available; a comparison would have been illuminating.

Reply: We believe analyses of methylation when TEs move to new positions requires more detail than can be provided in this paper. This comparison is now published, for families large enough to make statistical claims, by comparing insertion site occupancy in four genomes (B73, W22, Mo17, and PH207) in Noshay *et al.* (2019). We agree that it's very interesting, and further supports the idea that individual TE families can do many different things — bring methylation along, target methylated regions, and combinations of the two.

Reviewer Point P 1.27 — Line 415. Yes, because these are TEs that nucleate CHH islands.

Reply: Agree, but left this interpretation to the discussion.

Reviewer Point P 1.28 — Figure 6. Now the colors are no longer designated at all. Here and throughout, I would indicate right in the figure, above the data, what the superfamilies are. Also, since the point of this figure is, in general, to compare the TE and the flanking sequences, why not put B directly under A so that a visual comparison is possible. Frankly, although I accept the overall conclusions, with this and other figures I had trouble getting the point, other than that different families have differences in methylation in the TEs and their flanking sequences.

Reply: We've reduced this figure to focus on methylation of TEs and adjacent genomic regions. We hope that reducing the complexity to highlight the three methylation contexts improves clarity, although we agree a direct comparison of family-level variation is difficult (we just can't come up with a better way to display it!). Frankly, a takeaway of different families having different methylation in TEs and their flanking sequences is exactly what we were going for with this and other figures.

Reviewer Point P 1.29 — Line 413. This certainly seems to be true for some, but certainly not for all. Here and throughout some statistical support for what seems visually apparent would be helpful. Also, were the flanking sequences filtered for being TEs or not? What would TEs inserted into other TEs look like? What would families that are more likely to be inserted into other TEs look like.

Reply: We've elected to not differentiate copies between those inserted into non-TE sequence from those inserted into other TEs, simply because doing so doubles every analysis of the paper. Noshay *et al.* (2019) focuses on only non-nested copies, where a TE has inserted into non-TE sequence to truly tease apart these types of questions. We are presenting these patterns mainly for visual patterns, since the 2kb distance is mostly arbitrary.

We've added statistical support throughout the manuscript when we make comparisons between means or medians of different groups.

Reviewer Point P 1.30 — Line 431. Are these older elements where there was a lot of C to T conversion due to methylation?

Reply: Good question. We added a summary counting TG and CA dinucleotides within each TE, which would result from the deamination of C to T. Indeed, CG content and TG/CA content are negatively correlated, suggesting a tradeoff between these dinucleotides originating from deamination of methylated cytosines (Supp Figure **??**). For individual families, GC content is sometimes positively and sometimes negatively correlated with age (Figure 7C).

Reviewer Point P 1.31 — Line 477. Statistical support for a difference?

Reply: Yes, we performed pairwise Wilcoxon rank sum tests amongst TE orders to show that each order has significantly different expression of nearby genes. We also added information about median expression of nearby syntenic genes. Throughout, we've added statistical tests to support our claims.

Reviewer Point P 1.32 — Line 486. The maize genome is notorious for having large numbers of genes that are unlikely to actually be genes. I wonder what this analysis would look like if only syntenic genes were examined. I think this would be more informative.

Reply: We expect most of these lowly expressed genes to be non-syntenic genes. We agree that adding information about expression of nearby syntenic genes adds to our interpretation, and have included this for all tissues. As expected, subsetting by syntenic genes increases the distance of any TE to a gene (Supp. Figure 1B). Additionally, syntenic genes near TEs show higher expression, although orders are not significantly different in their median expression.

Reviewer Point P 1.33 — Line 494. This kind of makes sense, since it is likely that some elements insert into regions of relatively open chromatin and are thus more likely to be expressed.

Reply: Agreed, and it's hard to say whether that is a feature of the TE or of the gene or of the general genomic region.

Reviewer Point P 1.34 — Line 504. Interesting. Statistically significant?

Reply: Here we've added Wilcoxon rank sum test results that show a significant effect of subgenome.

Reviewer Point P 1.35 — Line 536. What about the effects of C to T mutations due to methylation?

Reply: Mutation rates are calculated as averages across nucleotide contexts, including the mutation rate we use (Clark *et al.*, 2005). While mutation rates may be higher in TEs than genes as they are in Arabidopsis (Weng *et al.*, 2018), probably due to deamination, it is beyond the scope of this paper to systematically address this. We apply a Kimura 2-Parameter correction to our LTR-LTR estimates of age, which does allow for unequal transition and transversion rates, and helps to correct for some of the effects of biased mutation on age.

Reviewer Point P 1.36 — Line 565. Is this because there are more likely to be more polymorphisms?

Reply: Yes, in general, older TEs should accumulate more nucleotide mutations. But this metric of segregating sites is summarizing single nucleotide mutations that have occurred within that TE copy across all resequenced individuals (who may or may not share this particular TE insertion). If a TE present in a single copy in the B73 reference genome were found in many copies in resequenced individuals, we would be unlikely to recover SNPs

within that TE, as most SNP calling uses hard cutoffs of mapping depth to avoid calling SNPs in highly repetitive regions. This could lead to old copies showing fewer segregating sites, since there would be so many reads mapping to the region that segregating site density is low.

Reviewer Point P 1.37 — Line 571. This is surprising, given what we know about selection against TEs near genes and the fact that CHH is mostly associated with TEs near genes.

Reply: Yes, I think this is part of the reason we shouldn't be thinking about these features in isolation. Likely the interaction between CHH methylation and gene distance combine to generate patterns of selection. The relationship between CHH and genes is discussed in more detail in the discussion.

Reviewer Point P 1.38 — Line 616. There are better, more recent references for this.

Reply: We added a references to more general recent reviews on the subject (Bennetzen *et al.*, 2005; Han *et al.*, 2013; Wicker *et al.*, 2016).

Reviewer Point P 1.39 — Line 631. This is reasonable, but what if selection acts more efficiently to remove large LTR insertions near genes?

Reply: One of the limitations of a single genome is that it's impossible to separate the interaction between insertion preferences and differential selection. We added a sentence at the end of this paragraph that highlights this fact. We're excited to begin to disentangle their effects as more and more genome assemblies become available.

Reviewer Point P 1.40 — Line 633. This is also supported by de novo insertion data.

Reply: Of course, great point. Added references for the abundant evidence of targeting of Mu insertions in maize.

Reviewer Point P 1.41 — Line 650. That is fascinating. I had no idea that families diversified so quickly. Then again, this depends on how families are defined.

Reply: Yes, we're using the commonly used 80% sequence identity cutoff, but even 50% sequence identity would result in a maximum of about 7 million years, which is still an age much younger than the divergence of maize and sorghum (12 million years).

Reviewer Point P 1.42 — Line 695. Awkward wording.

Reply: Agreed, changed 'numerous cases of insertional mutations in domestication and improvement genes' to 'the contribution of TE insertions to maize domestication and improvement.'

Reviewer Point P 1.43 — Line 726. Do you mean newly inserted?

Reply: Yes, in the process of inserting. Changed.

Reviewer Point P 1.44 — Line 732. Any evidence for that?

Reply: Cinful-zeon seems to be the best evidence. We've reworked the wording here to make it more explicit that we see mutualism as an analogy.

Reviewer Point P 1.45 — Line 738. Any evidence that Zeon and Cinful work together? This is an intriguing hypothesis, but it is speculative and should presented as such. Still, this a really intriguing idea!

Reply: Previous evidence has been limited to the presence of Cinful and Zeon arriving at similar times in the genome — it would take some rather ambitious experiments to support such a model (e.g. sequencing the cDNA molecules found within virus like particles during active transposition). We agree that such direct tests of some of these ecological analogies are really exciting, and hope to pursue them in the future, but they seem outside the scope of this manuscript.

Reviewer Point P 1.46 — Line 779. I'm not clear as to why this is not a somewhat trivial observation.

Reply: After adding in additional predictors, namely the proportion of TG dinucleotides in the sequence, and expression of the closest syntenic genes to each TE, the number of segregating sites is no longer the most predictive feature in the model. We've reduced this section in the manuscript. We agree that we expect to see more segregating sites with elevated age, and we do see that for most families (Figure 7C). There are some families where older copies show fewer segregating sites. We think this might be due to elevated coverage of read mapping to repetitive sequence that results in the site being removed during SNP calling. This would be particularly prevalent if members of the TE family have expanded in other maize genomes represented in HapMap3, but are low copy in B73. See an earlier response (Point 1.36) for more detail about this.

Reviewer Point P 1.47 — Line 813. As is evidenced by the very low recombination rates outside of genes in maize.

Reply: Yes, good point. Added a citation to a careful dissection of differential recombination in genic and retrotransposon regions around the bronze locus (Fu *et al.*, 2002).

Reviewer 2

This paper provides a detailed, rigorous description of the properties, abundances, distributions, and ages of transposable elements in the maize genome. The analyses are impressive and, in general, the paper is well-written (but see below for some significant issues). I think it will make a valuable contribution to the literature. However, I do have some comments for revision, which I present in the spirit of improving an already strong paper before publication.

Major points:

Reviewer Point P 2.1 — - I think the data presented in this paper are very useful, and the paper provides a very detailed overview of TE content in the maize genome. However, I struggle to see just how this is an example of "genome ecology" properly understood, for two reasons:

1) The paper does not actually use any methods from ecology. So, here — as in most previous uses of the concept — "ecology" serves as analogy rather than analysis. It's a bioinformatics study that describes TE properties and then layers on the ecological analogy. This is fine, but it may be worth noting that this is what is going on as opposed to, say, actually using ecological methods with genome data.

Examples of studies that are explicitly "genome ecology" in methodology and not just metaphor include:

Saylor et al. (2013). A novel application of ecological analyses to assess transposable element distributions in the genome of the domestic cow, Bos taurus. Genome 56: 521-533.

Serra et al. (2013). Neutral theory predicts the relative abundance and diversity of genetic elements in a broad array of eukaryotic genomes. PLOS One 8: E63915.

Linquist et al. (2015). Applying ecological models to communities of genetic elements: the case of neutral theory. Molecular Ecology 24: 3232-3242.

Reply: We agree that we are presenting a metaphor. Indeed, these papers shaped much of our thinking about applying ecological models to the genomic ecosystem. In our final versions, we removed the species rank abundance curves that were present in earlier drafts. We hope to introduce these methods in future studies. We've added these missed citations to our introduction, and added a section highlighting the difference between ecological and evolutionary approaches.

2) As noted by Linquist et al. (2013; Bio. Rev. 8: 573-584), most previous examples of "genome ecology" were actually "genome evolution". They used population genetics models or phylogenetics, focused on evolutionary timescales, involves evolution of TEs and genomes, and so on. This paper actually tends to drift into this category as well. Notably, most of the section on "The Family-level Ecology of the Genome" talks about evolutionary mechanisms and patterns; there is actually very little ecology discussed.

Reply: The distinction between ecology and evolution is difficult, especially for ecosystem engineers like TEs. We believe we're investigating local environmental factors that are allowing the survival of TEs — ecology — but can see how ecology can feed back to evolution to explain some patterns we describe. We've added text admitting our evolutionary bent in the 'Family-level ecology of the genome' section.

Reviewer Point P 2.2 —

- The Introduction is very well written, and it is refreshing to see a nuanced overview of TEs rather than the trope of "long dismissed as junk DNA...". Where the paper needs work is in the Results and Discussion. Here, the paper suffers from what is sometimes called "thesis syndrome", where a paper for publication reads very much like a graduate thesis chapter.

1) Results — This section contains way too much material that should be in the Discussion (or Introduction). Interpretations of findings, reviews of previous literature, etc., do not belong in the Results section. Here, the findings should be presented without interpretation, allowing the reader to agree with or challenge the patterns reported independently of whether they agree with your interpretation.

Reply: We have reduced descriptions and interpretations in the results. We didn't remove all citations and explanations, namely leaving those that help guide the reader towards why we are presenting certain results.

2) Discussion — This section spends far too much time discussing the work of others. The Discussion in a data paper (versus a review paper) should discuss your results first and foremost. Citing the literature should mostly be done to put your findings in broader context (after you have discussed them, not "so and so said... and we found that too") and/or to back up an interpretation that you wish to present or a claim you are making. Major sections of the Discussion are more like a review paper than a discussion of the results in a data paper.

Reply: We've rearranged the presentation of discussion material to highlight our findings first, and then put them into context afterwards.

Minor comments:

Reviewer Point P 2.3 — - Despite what elementary school students are taught, there is no specific grammatical rule that forbids the use of "But" to start a sentence. However, doing so 10 times in a single paper is excessive and inelegant (the tendency to over-use it is probably why elementary school students are told not to do it at all).

Reply: We've removed all but one usage of this conjunction to start a sentence.

Reviewer Point P 2.4 — - p.7 — "(Figure 2; Interactive distributions per family:)." Not sure what this is supposed to say.

Reply: The link didn't render properly, we've added the link in the revised version.

References

Anderson, S. N., M. C. Stitzer, P. Zhou, J. Ross-Ibarra, C. D. Hirsch, *et al.*, 2019 Dynamic patterns of transcript abundance of transposable element families in maize. G3: Genes, Genomes, Genetics .

Anderson, S. N., G. Zynda, J. Song, Z. Han, M. Vaughn, *et al.*, 2018 Subtle perturbations of the maize methylome reveal genes and transposons silenced by chromomethylase or rna-directed dna methylation pathways. G3: Genes, Genomes, Genetics pp. g3–200284.

Bennetzen, J., R. Liu, J. Ma, and A. Pontaroli, 2005 Maize genome structure and rearrangement. Maydica **50**: 387.

Bennetzen, J. L., 2000 Transposable element contributions to plant gene and genome evolution. Plant Molecular Biology **42**: 251–269.

Clark, R. M., S. Tavaré, and J. Doebley, 2005 Estimating a nucleotide substitution rate for maize from polymorphism at a major domestication locus. Molecular biology and evolution **22**: 2304–2312.

Fu, H., Z. Zheng, and H. K. Dooner, 2002 Recombination rates between adjacent genic and retrotransposon regions in maize vary by 2 orders of magnitude. Proceedings of the National Academy of Sciences **99**: 1082–1087.

Han, Y., S. Qin, and S. R. Wessler, 2013 Comparison of class 2 transposable elements at superfamily resolution reveals conserved and distinct features in cereal grass genomes. BMC genomics **14**: 71.

Inouye, S., S. Yuki, and K. Saigo, 1984 Sequence-specific insertion of the drosophila transposable genetic element 17.6. Nature **310**: 332.

Noshay, J. M., S. N. Anderson, P. Zhou, L. Ji, W. Ricci, *et al.*, 2019 Monitoring the interplay between transposable element families and dna methylation in maize. PLoS genetics **15**: e1008291.

SanMiguel, P., A. Tikhonov, Y.-K. Jin, N. Motchoulskaia, *et al.*, 1996 Nested retrotransposons in the intergenic regions of the maize genome. Science **274**: 765.

Schnable, P. S., D. Ware, R. S. Fulton, J. C. Stein, F. Wei, *et al.*, 2009 The B73 maize genome: complexity, diversity, and dynamics. science **326**: 1112–1115.

Sultana, T., A. Zamborlini, G. Cristofari, and P. Lesage, 2017 Integration site selection by retroviruses and transposable elements in eukaryotes. Nature Reviews Genetics **18**: 292.

Washburn, J. D., M. K. Mejia-Guerra, G. Ramstein, K. A. Kremling, R. Valluru, *et al.*, 2019 Evolutionarily informed deep learning methods for predicting relative transcript abundance from dna sequence. Proceedings of the National Academy of Sciences **116**: 5542–5549.

Weng, M.-L., C. Becker, J. Hildebrandt, M. T. Rutter, R. G. Shaw, *et al.*, 2018 Fine-grained analysis of spontaneous mutation spectrum and frequency in arabidopsis thaliana. Genetics pp. genetics–301721.

Wicker, T., Y. Yu, G. Haberer, K. F. Mayer, P. R. Marri, *et al.*, 2016 Dna transposon activity is associated with increased mutation rates in genes of rice and other grasses. Nature communications **7**: 12790.

Yuan, Y.-W. and S. R. Wessler, 2011 The catalytic domain of all eukaryotic cut-and-paste transposase superfamilies. Proceedings of the National Academy of Sciences **108**: 7884–7889.
