# Supplementary material for: The genomic ecosystem of transposable elements in maize
Source: PLoS Genet. 2021 Oct 14;17(10):e1009768. doi: 10.1371/journal.pgen.1009768 (PMC8547701; doi:10.1371/journal.pgen.1009768)
Supplement: Attachment — Submitted filename: response_to_second_review.pdf [file pgen.1009768.s022.pdf]

From the editor:

Thanks for your edits! The paper is much improved. The reviewer however makes some remaining comments and I think that some of these are worth addressing. So I am giving this a "minor revision" - it will not need to go back out for review after these changes. Thanks!

Reviewer 1

Reviewer Point P 1.1 — Please note that I think the manuscript is fine as it is (although I do think some aspects of figure 7 could be clear). My comments below are simply meant to provide food for thought.

Reply: Thank you!
We flipped the Figure 7B with Figure 7C to hopefully associate the labels to both, and added text to the figure legend reflecting that labels belong to both B and C.

Reviewer Point P 1.2 — Line 176. I think I get what the authors are getting at here, but it occurs to me from the text here, as well as the other reviewers comments, that I'm actually not entirely clear as to the distinction between ecological and evolutionary processes. That's probably just because I'm not a specialist in either field, but what exactly is the distinction. In this context, does "ecological" mean the interaction between different TEs and between TEs and their hosts? I'm assuming here that it does not mean the affects that TEs have on ecological processes occurring at the level of the host. Does it also mean interactions that have an impact on survival of a given TE lineage? A google definition (sorry, I mentioned that I'm not a specialist) of niche is "all of the interactions of a species with the other members of its community, including competition, predation, parasitism, and mutualism. A variety of abiotic factors, such as soil type and climate, also define a species' niche." What are the analogies here? Taking a gene-centric view, I suppose each group of TEs that can cross mobilize constitute a "species", and TEs "speciate" when one lineage gives rise to two lineages that can no longer cross mobilize. Individuals would be individual stretches of DNA that can replicate (TEs), or can contribute to host function (host genes). However, with TEs get more squirrely, since any give insertion is, in situ, "alive" only to the extent that it retains the capacity to replicate, which is a function of its particular niche. Or something like that. Ecological functions would have to do with all of the particular features that would allow a given TE (or, more properly, TE lineage) to thrive over time, as well as the impact that this TE has on both the other TEs and on host fitness. In contrast, Evolutionary process would be what? Time scale? Neutral processes that result in large changes in genome architecture that are random with respect to ecological processes (at the genome level)? Would this be an example of the distinction? Some TEs target heterochromatin (and thus often other TEs) and some targe TSSs by tethering to components of the POLII complex. These two distinct strategies represent "niches", with both costs a benefits, so this is Ecological. In contrast, Gout has shown that selection over time tends to remove TEs from regions immediately adjacent to genes, so TEs near genes tend to be younger. So if you have one TE family that is older than a second family, the older family will be distributed differently than the younger family even if the TEs have exactly the same targeting initially. These TEs do not occupy distinct "niches"; the differences are purely a function of selection at the level of the host and time. So in figure 3, if age were mapped on to these distributions, you might find a correlation between age and presence in the pericentromeres (CACTAs are, as I recall older and found more frequently in these regions). Similarly, one can imagine a variety of stochastic events that ends up favoring a given TE family that have nothing to do with particular strategies employed by a given TE that impact distribution. Horizontal transfer, for example, that moves a TE from a highly suppressive environment to a more permissive environment. These would be "Evolutionary" processes. Please forgive me for going on, but I'm just trying to give the authors a sense of what comes up for me when reading the text. Hopefully it is of some use to the authors.

Reply: This is great and really helpful. To me, ecology represents the interactions between individuals and of individuals with their environment, while evolution investigates change of a population of individuals through time. We struggle in the genome with the distinction of evolutionary and ecological processes, especially with recent advances in the realm of eco-evo feedbacks. The two are difficult to separate, because they do exist on a continuum and are shaped by each other (although I think reflecting the same underlying biological reality).

For the genomic ecosystem, generations get a little tricky - which to me is where the crux of the breakdown of the metaphor is. I think of ecology as the interactions that occur between TEs, TE families, and their host genome that exist in the extant genome, as we've sampled it in B73. Now these interactions are shaped by evolution through time, which to me is enforced by generations of the host.

We feel that these distinctions are important, but outside the scope of this first paper - there's lots of details of these interactions that need to be followed up in depth. These reviewer comments help to clarify how to approach presenting these ideas!

Reviewer Point P 1.3 — Line 208. Thank you for not using the word "respectively" :)

Reply: So many acronyms!

Reviewer Point P 1.4 — Line 246. Is "years ago" correct? Is the median age of people in nursing homes 82 years ago?

Reply: Good point, removed "ago."

Reviewer Point P 1.5 — Line 267. Good call. Since plant genomes have such a high turnover rate, even if a given lineage did "arrive in the genome" a very long time ago, we could not know when.

Reply: Agreed.

Reviewer Point P 1.6 — Line 299. This makes sense, as the copy number of older families, and the overall number of families as defined would go up as time passed. So if you started with a massive bloom and 10,000 copies, all of which were DOA, then, as copies were deleted and the sequences diverged to about 80% similar, you would end up with hundreds of distinct "families", each with a very low copy number.

Reply: Yes, unsuccessful, diverged copies exist. This is another thing we can start to test with more and more genome assemblies - old copies should be shared at the same position among individuals.

Reviewer Point P 1.7 — Line 303. Both the TIR and helitron examples make me think about gene capture. In rice, there are about 3000 Pack Mules, the average copy number of each of which is about two. Because they do not share the same internal sequences, they could be placed within different families (as defined), but many of them could be the result of capture events mediated by a particular autonomous MULE, which could theoretically mobilize them. Mu elements in maize are a clear example of this. The autonomous element has high homology at the TIRs with the non-autonomous elements but none in the internal sequences. Nevertheless, when the autonomous element is active, all of the non-autonomous elements can replicate. So I'm not sure if they are part of the same "species" or not. Or perhaps the non-autonomous elements can be thought of as parasites? If so, then how does this fit into an ecological model?

Reply: Ideally, families/species would completely recapitulate the autonomous/nonautonomous relationship between TE copies (and somehow also do this through time). We're including what seem to be nonautonomous copies, since 80% of the shorter (nonautonomous) copy matches with 80% identity to an autonomous copy, they get joined in one family. We're also including potential gene captures, where 80% of the shorter (without additional gene sequence) copy matches with 80% identity to the longer captured copy. While this is imperfect, it does in some way connect these extreme examples into a family, as long as there are enough family members in the genome to connect them up. There's a school of ecological thought that competition within species has to be stronger than that between species to allow persistence of multiple species, which has been shown experimentally for fly TEs, e.g. Robillard *et al.* (2016).

We hope that future experimental approaches in maize help to address true interactions between members of a family defined by sequence similarity.

Reviewer Point P 1.8 — Figure 4. What really jumps out for me are two things. First, many of the superfamilies appear to have experience big jumps in copy number recently. Is this really a thing? Second, it really looks like the key distinction with respect to age is at the Family, not the superfamily level. Interesting.

Reply: Yes, we see what looks like a recent accumulation, especially in DNA transposons. This might be conflated with actual mechanism of movement (retrotransposition by a non-proofreading reverse transcriptase is likely to generate more mutational differences than excision of a TIR DNA transposon). The differences in ages of families is part of what makes me believe them at least a little bit, as it argues against consistent methodological issues pushing age estimates towards the present. Again, looking at multiple maize individuals will help with knowing how much recent transposition is actually going on.

Reviewer Point P 1.9 — Line 368. A quibble, but saying "partition" suggests that this is a distribution derived from functional constraints rather than a consequence of random loss of function within multiple copies.

Reply: Changed 'partition' to 'is split between.'

Reviewer Point P 1.10 — line 501. A median of zero is just zero, right?

Reply: There are copies with greater than 0 methylatable cytosines, it's just so few copies that the median is still 0.

Reviewer Point P 1.11 — Line 508. Again, not for this publication, but I wonder if the proportion of converted cytosines in the CG and CHG versus tell a history about a given element. CHH islands tend to be near genes, so a TE lineage that has spent a lot of time near genes could have a different proportion than a TE lineage that didn't.

Reply: I feel like they must. If transpositional activity is maintained, it seems like a great strategy for a TE to get completely deaminated and then only have post-transcriptional gene silencing. However, sometimes I think about how complicated methylation needs to be for something like Ac transposase to bind (just one TIR/end needs to be methylated, if both are, excision doesn't happen). Selection vs. accident will be fun to disentangle.

Reviewer Point P 1.12 — Line 550. "...by family, and for example the two.." awkward

Reply: Changed to "varies by family – the two largest families."

Reviewer Point P 1.13 — Line 562. One wonders if this is because many of the "genes" near helitrons are actually transduplicated parts of the helitron.

Reply: Yes, probably. This is supported by the observation that the closest syntenic gene to helitrons is more highly expressed (Supp. Figure 1F,G,H), aka that these nonsyntenic gene bits nearby aren't really genes.

Reviewer Point P 1.14 — 567. Again, I'm probably just being thick, but a median of zero means half were above zero and half were below (which they can't be), so none of them expressed at all?

Reply: I think it's the middle of the ranked list, so all below are 0, some above are greater than zero.

Reviewer Point P 1.15 — Figure 6. In legend. D-F?

Reply: I see, rearranged to get labels back to alphabetical order.

Reviewer Point P 1.16 — Line 635. That's a bit surprising given figure 4, which suggests that there is more variation at the family level.

Reply: Yes, it's a little bit of nuance, but knowing superfamily tells you a lot about what ages to expect. For example, if I told you you were a DTT superfamily, most of the time age would be 0.

Reviewer Point P 1.17 — Line 655. It's not clear what each line on the Y axis represents here. Correlates with the Y axis names to the left? F-G. what do the colors correspond to? The colors of the labels of families in C? I'm afraid these need a bit more explanation in the legend.

Reply: I see. We flipped the x axis to better associate labels with bars.

Reviewer Point P 1.18 — Line 747. And by analysis of de novo, unselected insertions.

Reply: Yes, new insertions will solve this.

Reviewer Point P 1.19 — Line 779. See also https://bmcgenomics.biomedcentral.com/articles/10.1186/s12864-020-6763-1

Reply: We've added a citation to this recent paper earlier in the introduction, and later in the discussion.

Reviewer Point P 1.20 — Line 794. See my comment earlier about partitioning.

Reply: Changed 'partitioning' to 'encode different proteins required for retrotransposition.'

Reviewer Point P 1.21 — Line 952. "elaborate genome" :)

Reply:

References

Robillard, É., A. Le Rouzic, Z. Zhang, P. Capy, and A. Hua-Van, 2016 Experimental evolution reveals hyperparasitic interactions among transposable elements. Proceedings of the National Academy of Sciences **113**: 14763–14768.
